# Synthesis of cuprous oxide/silver (Cu_2_O/Ag) hybrid as surface-enhanced Raman scattering probe for trace determination of methyl orange

**DOI:** 10.1098/rsos.221623

**Published:** 2023-05-24

**Authors:** Thi Thu Ha Pham, Xuan Hoa Vu, Nguyen Dac Dien, Tran Thu Trang, Nguyen Van Hao, Nguyen Duc Toan, Nghiem Thi Ha Lien, Tong Sy Tien, Tran Thi Kim Chi, Nguyen Thi Hien, Pham Minh Tan, Dong Thi Linh

**Affiliations:** ^1^ Faculty of Chemistry, TNU-University of Sciences, Tan Thinh ward, Thai Nguyen city 24000, Vietnam; ^2^ Institute of Science and Technology, TNU-University of Sciences, Tan Thinh ward, Thai Nguyen city 24000, Vietnam; ^3^ Faculty of Occupational Safety and Health, Vietnam Trade Union University, 169 Tay Son street, Dong Da district, Ha Noi city 100000, Vietnam; ^4^ Centre for Quantum Electronics, Institute of Physics, Vietnam Academy of Science and Technology, 18 Hoang Quoc Viet road, Cau Giay district, Ha Noi city 100000, Vietnam; ^5^ University of Fire Prevention and Fighting, 243 Khuat Duy Tien road, Thanh Xuan district, Ha Noi city 100000, Vietnam; ^6^ Institute of Materials Science, Vietnam Academy of Science and Technology, 18 Hoang Quoc Viet road, Cau Giay district, Ha Noi city 100000, Vietnam; ^7^ Faculty of Fundamental Sciences, Thai Nguyen University of Technology, 666 3/2 road, Thai Nguyen city 24000, Vietnam

**Keywords:** metal–semiconductor composite, surface-enhanced Raman scattering, cuprous oxide, silver nanoparticles, methyl orange

## Abstract

Recently, there have been publications on preparing hybrid materials between noble metal and semiconductor for applications in surface-enhanced Raman scattering (SERS) substrates to detect some toxic organic dyes. However, the use of cuprous oxide/silver (Cu_2_O/Ag) to measure the trace amounts of methyl orange (MO) has not been reported. Therefore, in this study, the trace level of MO in water solvent was determined using a SERS substrate based on Cu_2_O microcubes combined with silver nanoparticles (Ag NPs). Herein, a series of Cu_2_O/Ag*x* (x= 1–5) hybrids with various Ag amounts was synthesized via a solvothermal method followed by a reduction process, and their SERS performance was studied in detail. X-ray diffraction (XRD) and scanning electron microscopy results confirmed that 10 nm Ag NPs were well dispersed on 200–500 nm Cu_2_O microcubes to form Cu_2_O/Ag heterojunctions. Using the as-prepared Cu_2_O and Cu_2_O/Ag*x* as MO probe, the Cu_2_O/Ag5 nanocomposite showed the highest SERS activity of all samples with the limit of detection as low to 1 nM and the enhancement factor as high as 4 × 10^8^. The logarithm of the SERS peak intensity at 1389 cm^−1^ increased linearly with the logarithm of the concentration of MO in the range from 1 nM to 0.1 mM.

## 1. Introduction

Since its discovery in 1974 [1], the surface-enhanced Raman scattering (SERS) technique has been extensively studied because of its characteristics of significant amplification of the Raman intensity of analytes adsorbed on substrate surface [2–4]. Due to the development of material surface science and nanotechnology, the SERS technique has developed rapidly in recent decades [5,6]. Owing to its accuracy and reliability, SERS is a rapid, *in situ*, non-destructive and ultrasensitive analytical tool [7,8]. SERS can detect the trace level of target molecules adsorbed on the surface of the metal or semiconductor substrate with limit of detection (LOD) in the concentration range of nanomole per litre (nM) [9–11]. The interaction between analytes and substrates generates Raman signals which are influenced by the material and morphological characters of the substrate. The sensitivity, recovery and reproducibility are important parameters of SERS substrates [12]. As we have known, electromagnetic mechanism (EM) and chemical mechanism (CM) are the two main mechanisms for SERS enhancement. In EM mechanism, surface plasmon resonance (SPR) provides a large electromagnetic field to enhance Raman signal. In CM mechanism, charge transfer (CT) between SERS substrate and adsorbed molecules increases the SERS intensity [13]. The combination of noble metals (Ag and Au) and semiconductor materials (TiO_2_, Fe_3_O_4_, Cu_2_O, SiO_2_, ZnO, SnO_2_ and CuO) [14–20] gave significantly enhanced SERS signals because of the synergy of EM and CM [21,22]. Ag is one of the noble metals commonly used for SERS substrate owing to its strong localized SPR (LSPR) and narrow plasmon bandwidth in the visible region [23,24]. The combination of noble metal and semiconductor remarkably improves the practical application ability of SERS substrate [25]. For instance, Xu *et al*. used Au/ZnO nanorods to detect rhodamine 6G (R6G) molecules with a low LOD of 10^−9^ M [26]. He *et al*. obtained urchin-like silver nanoparticle (Ag NP)/ZnO hollow nanosphere arrays for the detection of R6G molecules with great SERS performances (enhancement factor (EF) of 10^8^ and LOD of 10^−10^ M) [27].

Cuprous oxide (Cu_2_O) is a p-type semiconductor metal oxide with a direct band gap of 2.17 eV [28] as a potential material in catalysis and sensing performance [16,29]. The narrow band gap allows Cu_2_O to generate active electron–hole pairs by visible light [30]. Cu_2_O has been widely used in photocatalysis [31], electrochemistry [32,33], photovoltaic conversion [29], dye bleaching [34], sterilization [10] and SERS [5,9,15]. In recent years, there are publications on the synthesis of Cu_2_O nanostructures, such as nanoflowers [5], nanoparticles [10,33,35,36], nanocubes [16], sub-micro cubes [37], octahedra [12,38], dodecahedra [25], polyhedra [39], nanowires [40], microspheres [41,42], nanospheres [32] and chestnut-like Cu_2_O [43]. When Ag NPs adhere to the surface of Cu_2_O, the CT between two materials will occur under appropriate irradiation [44]. The high charge density produces a strong LSPR enhancing electromagnetic field [45]. Although the SERS activity of pure Cu_2_O nanomaterials is relatively poor, Cu_2_O/Ag composite can produce excellent SERS activity that has been confirmed by many studies. For example, Chen *et al*. [12] synthesized octahedral Cu_2_O-Au composite microstructures to detect 4-mercaptobenzoic acid. Liu *et al*. prepared Cu_2_O/Ag composite film that exhibits good performance as a SERS substrate for detecting various molecules such as rhodamine B (RhB), methylene blue and R6G [44]. Jiao *et al*. obtained Cu_2_O nano-octahedrons/Ag nanovines heterostructures and applied them in detecting thiram molecules [38], and so on. When the target molecules anchor to Cu_2_O substrate, they can be effectively and selectively identified by the semiconductor. Basing on the investigation above, we supposed that the Cu_2_O/Ag binary composite would exhibit enhanced SERS performance. Here, Cu_2_O microcubes were prepared via a solvothermal method followed by chemical reduction to load Ag nanoparticles on the surface of Cu_2_O microcubes, and their SERS performances were investigated in detail.

**Figure 1.**
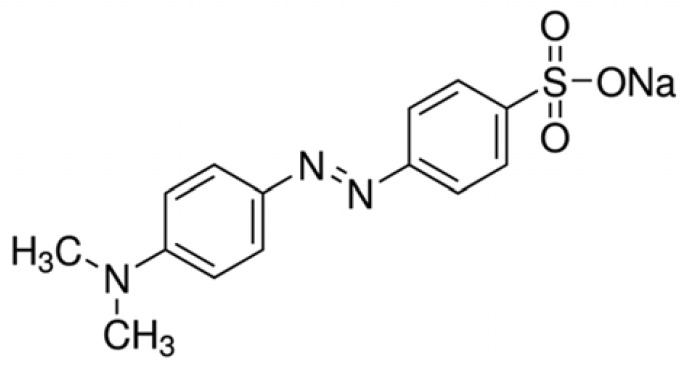
Molecular structure of MO.

Methyl orange (MO) (dimethylaminoazobenzenesulfonate) is a common and typical azo anionic, water-soluble organic synthesis dye. Owing to its high colourability and bright orange colour in water, it is used to dye several fabrics, including cotton, silk, polyester and nylon. Its molecular formula (C_14_H_14_N_3_NaO_3_S) contains aromatic and − *N* = *N* − groups which are highly toxic, carcinogenic and teratogenic to organisms (figure 1).

Therefore, it is categorized as an acute toxic compound and mutagenic substance [46]. The abuse of MO in food industry and improper discharging it into wastewater will cause the residue of toxic substance leading to increasingly environmental pollution. It affects the habitat of aquatic environments and produces different amines under anaerobic conditions [47]. Therefore, developing new-generation SERS substrates that are effective, sensitive, easy to fabricate, environmentally friendly and low-cost to monitor MO in wastewater is an urgent issue. Recently, Van Nguyen *et al*. used silicon integrated with Ag nanoparticles to detect MO with the LOD of MO as 10^−9^ M and the linear range from 10^−9^ to 10^−3^ M [48]. Zarei and co-workers have embedded Ag nanoparticles in diamond-like carbon, which gave the EF of 2.9 × 10^8^ toward 6 mM of MO [49]. Si and colleagues used Ag colloids as SERS substrate to measure the concentration of MO [50]. However, to the best of our knowledge, few studies on detecting MO using SERS method have been published, and this is the first publication providing a novel approach to determine MO using Cu_2_O microcubes/Ag NP-based SERS substrate. In this research, a series of Cu_2_O microcubes/Ag nanoparticles hybrids with different amounts of Ag was synthesized via solvothermal and reduction processes. The as-prepared Cu_2_O/Ag*x* samples were used as SERS probe with 532 nm laser excitation for the determination of trace MO. They showed a good property in terms of adsorption capacity, SERS sensitivity and selectivity. We obtained different Raman spectra by adjusting the MO concentration. The SERS spectra had information on the CT process between Cu_2_O/Ag*x* and MO molecules. In addition, small specific surface area of Ag NPs and heterostructure of Cu_2_O/Ag had a synergistic effect on the SERS signal.

## 2. Experimental

Copper sulfate (CuSO_4_), sodium hydroxide (NaOH), ethylene glycol (EG) (CH_2_OH)_2_ or C_2_H_4_(OH)_2_, hydrazinium hydrate (N_2_H_4_·H_2_O), silver nitrate (AgNO_3_) and sodium borohydride (NaBH_4_)—all of analytical grade—were purchased from Sigma-Aldrich and applied as received without further purification. CuSO_4_ was used as the precursor of Cu_2_O, and AgNO_3_ was used for Ag preparation. The obtained Cu_2_O and Ag were supplied to synthesized Cu_2_O/Ag hybrids. MO was used as the analyte. Deionized water was used to prepare all aqueous solutions and clean glasswares.

The synthesizing process of Cu_2_O microcubes was as follows: first, 6 g NaOH was dissolved in 30 ml of deionized water, stirred for 15 min until the NaOH was completely dissolved, then 8 g CuSO_4_ and 120 ml EG solution (as a stabilizing agent) were added into the NaOH solution under vigorous magnetic stirring at room temperature.

2.1 $${\mathrm{CuSO}}_{4}+2\mathrm{NaOH}\to \mathrm{Cu}{\left(\mathrm{OH}\right)}_{2}+{\mathrm{Na}}_{2}{\mathrm{SO}}_{4}.$$

In the alkaline medium, EG combined cupric ions to form a blue complex,

2.2 $$2C_{2}H_{4}{\left(\mathrm{OH}\right)}_{2}+\mathrm{Cu}{\left(\mathrm{OH}\right)}_{2}\to {\left(C_{2}H_{4}\mathrm{OOH}\right)}_{2}\mathrm{Cu}+2H_{2}O.$$

Then, 25 ml of N_2_H_4_ (0.5 M) solution was added drop by drop into the above-mentioned dispersion under continuous stirring for 1.5 h. In this reaction system, hydrazinium was used as a reductant to reduce Cu^2+^ ions to Cu^+^ and generate Cu_2_O crystal nucleus,

2.3 $$4{\left(C_{2}H_{4}\mathrm{OOH}\right)}_{2}\mathrm{Cu}+N_{2}H_{4}+2H_{2}O\to 2Cu_{2}O+\;N_{2}↑+8C_{2}H_{4}{\left(\mathrm{OH}\right)}_{2}.$$

N_2_H_4_ was gradually added to avoid the aggregation of Cu_2_O to deposit.

The obtained mixture was then transferred into a 200 ml Teflon-lined stainless steel autoclave and solvothermally treated at 180°C for 10 h in an electric oven to form Cu_2_O nanocrystallines. The formed Cu_2_O crystal nucleus and small Cu_2_O nanoparticles accelerated the growth rate of Cu_2_O nucleus in the three orientations to form Cu_2_O microcubes. After naturally cooling down to room temperature, the red precipitate was separated from the solution and rinsed three times with deionized water and ethanol via repeated redispersion and filtration. The samples were then dried at 80°C overnight.

In a typical Cu_2_O/Ag hybrid preparation, 0.1 g of as-synthesized Cu_2_O was dispersed into 50 ml of deionized water at room temperature through magnetic stirring. Then, 50 µl AgNO_3_ (0.2 M) solution was primarily mixed with Cu_2_O suspension with quick magnetic stirring in an ice water bath. Subsequently, 150 µl NaBH_4_ (0.2 M) solution as a reducing agent was slowly added into the above mixture under vigorously magnetic stirring for 10 min. The colour of the mixture turned to lemon yellow, indicating the appearance of metallic silver seeds as the below reaction,

2.4 $$2\mathrm{AgN}O_{3}+2\mathrm{NaB}H_{4}\to 2\mathrm{Ag}↓+2\mathrm{NaN}O_{3}+2BH_{3}+H_{2}↑.$$

Next, to uniformly disperse the Ag NPs onto the surface of the Cu_2_O microcubes, the mixture was placed in an ultrasonic cleaner for 10 min. The obtained precipitate was rinsed with ethanol for three times and dried at 80°C overnight in a vacuum oven. The as-prepared Ag NPs modified-Cu_2_O microcubes were called Cu_2_O/Ag1.

To control the silver amount on the surface of Cu_2_O, the Cu_2_O amount was kept constant (0.1 g), and the concentration of AgNO_3_ and NaBH_3_ was fixed as 0.2 M; the experiment was repeated with various volumes of AgNO_3_ solution (50, 150, 200, 300 and 400 µl). After gentle stirring, the corresponding volumes of NaBH_3_ solution were gradually added to the AgNO_3_-Cu_2_O mixed solution following table 1. The obtained products symbolized as Cu_2_O/Ag*x* (*x* = 1, 2, 3, 4, 5) were filtered, washed with absolute ethanol and deionized water several times, then dried at 80°C overnight in the ambient environment. The surface morphology of the samples was measured by a scanning electron microscopy (SEM, Hitachi S4800, Japan) operated at 80 kV acceleration voltage. The elemental composition of samples was examined via energy-dispersive X-ray spectroscopy (EDS) in a Hitachi SU 8020 at an accelerating voltage of 200 kV. Fourier transform infrared (FTIR) spectra in the scanning range of 400–4000 cm^−1^ were collected on a JASCO 4600 spectrophotometer (Japan). The X-ray diffraction (XRD) patterns were obtained on X-ray diffractometer (Bruker D8 Advance, Germany) equipped with a monochromatic Cu-K*_α_* radiation source (wavelength of 1.54056 Å) and set to 40 kV and 30 mA. Diffractograms were recorded at a 2 degree min^−1^ scanning rate from 20 to 80 two theta degrees. The ultraviolet–visible–near infrared (UV-Vis-NIR) absorption spectra were acquired on a JASCO V-770 spectrophotometer in the glass cuvette with a path length of 1 cm over the wavelength range of 250–1000 nm. Photoluminescence (PL) spectra were obtained from a fluorescence spectrophotometer (Hitachi F-7000, Japan). For SERS measurements, 10 mg of each sample (Cu_2_O, Cu_2_O/Ag*x* nanocrystal) was dispersed in 20 ml of deionized water, then suspensions dropped on a circular glass slide (diameter 5 mm) and dried naturally at room temperature to obtain a thin film. Using a micro Raman system, the positions chosen to collect SERS spectra were fully covered, allowing the signal caused by the glass slide to be ignored. After diluting the MO solution to achieve the desired concentrations ($10^{-4}$–10^−9^ M), 2 µl of each MO solution was deposited on the SERS substrates and dried at room temperature. The Raman spectra in this study were collected using Raman spectrometer (Raman Horiba XploRa plus Raman microprobe, France) under laser excitation of 532 nm. The laser power is kept at 3.2 mW, the attenuation is 1% and the accumulation time is 8 s. The diameter of the laser spot was 1 µm. At least five Raman measurements were obtained for each sample to verify spectral reproducibility.

**Table 1.** Cu_2_O/Ag composite with different amounts of Ag NPs.

no.	sample name	weight of Cu_2_O (g)	volume of AgNO_3_ (0.2 M) (µl)	volume of NaBH_4_ (0.2 M) (µl)
1	Cu_2_O/Ag1	0.1	50	125
2	Cu_2_O/Ag2	0.1	100	250
3	Cu_2_O/Ag3	0.1	200	500
4	Cu_2_O/Ag4	0.1	300	750
5	Cu_2_O/Ag5	0.1	400	1000

## 3. Results and discussion

### 3.1. Characterization of the synthesized materials

**Figure 2.**
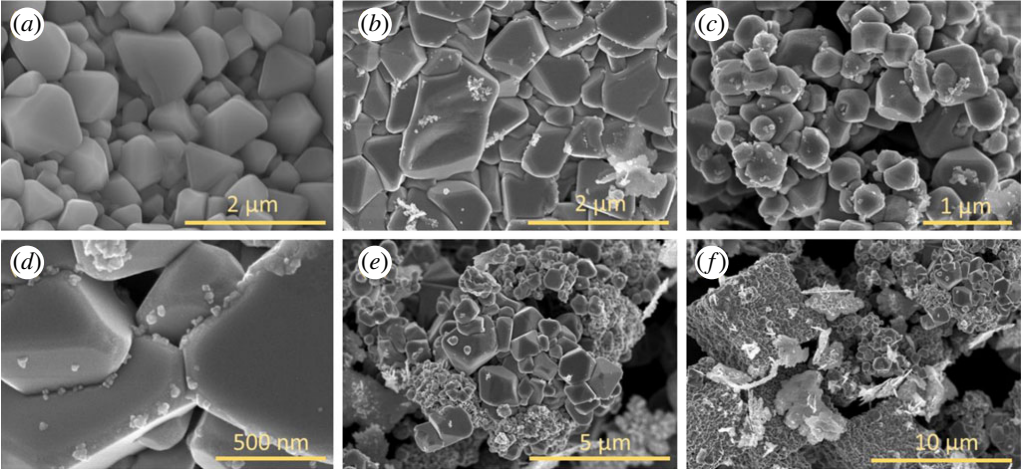
SEM images of (*a*) Cu_2_O, (*b*) Cu_2_O/Ag1, (*c*) Cu_2_O/Ag2, (*d*) Cu_2_O/Ag3, (*e*) Cu_2_O/Ag4 and (*f*) Cu_2_O/Ag5.

SEM images of Cu_2_O and Cu_2_O/Ag with different Ag amounts are presented in figure 2. Cubic Cu_2_O with the size of 0.5–1 µm was formed before adding Ag precursor (figure 2*a*). Following the addition of AgNO_3_ solution, a few Ag NPs of approximately 15–20 nm were tightly attached to the surface of Cu_2_O microcubes (figure 2*b*). When increasing the volume of AgNO_3_ solution, the coverage density of Ag NPs on Cu_2_O cubes increased (figure 2*c–e*).

In the case of Cu_2_O/Ag5 samples, the surface of Cu_2_O cubes was almost completely covered (figure 2*f*), which considerably increased the contact probability between target molecules and Ag NPs as well as preferred to the hot spot formation. Therefore, the SERS activity of this sample is expected to be better than the others, as observed in similar material classes, such as iron-silver nanoparticles (Ag NPs) sample [51] or Ag/Al_2_O_3_ [52]. As known from the literature, small crevices between nanoparticles support the optically induced electromagnetic field and the Raman cross-section enlargement, thus, improve the SERS signal intensity [43].

**Figure 3.**
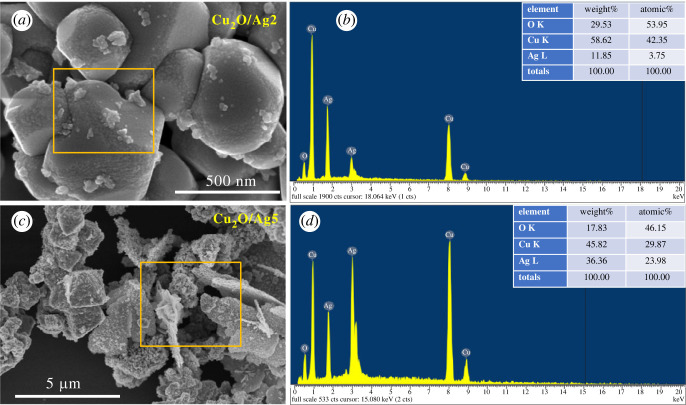
SEM image and EDS spectrum of (*a,b*) Cu_2_O/Ag2 and (*c,d*) Cu_2_O/Ag5.

**Figure 4.**
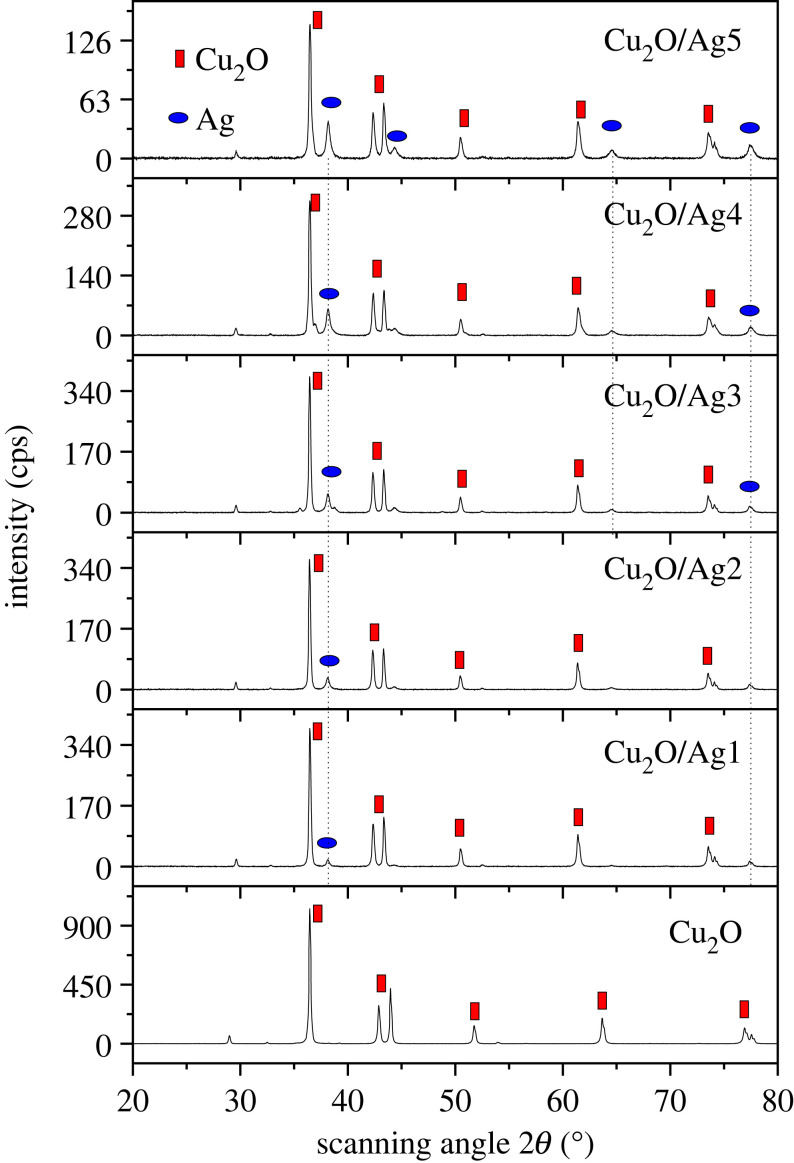
XRD patterns of Cu_2_O and Cu_2_O/Ag*x* (*x* = 1, 2, 3, 4, 5) samples.

**Figure 5.**
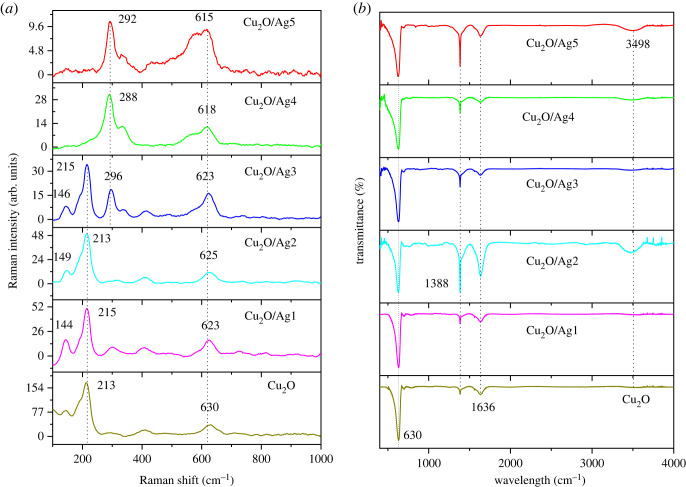
(*a*) Raman spectra and (*b*) FTIR spectra of Cu_2_O and Cu_2_O/Ag*x*.

The morphology of Cu_2_O/Ag2 and Cu_2_O/Ag5 samples was further characterized by high-resolution SEM images in figure 3*a,b*. There was clearly cuboid Cu_2_O with Ag NPs uniformly dispersed on their surfaces to form heterogeneous interface between Cu_2_O and Ag. Consequently, this junction could provide a synergistic effect on SERS activity. The energy-dispersive EDS data can identify the chemical composition of samples in detail. The EDS spectra of Cu_2_O/Ag2 and Cu_2_O/Ag5 in figure 3*b,d* show Cu peaks at 1, 8 and 9 keV; O peak at 0.5 keV, and Ag peaks at 1.8 and 2.9 keV, confirming that the samples contained three elements (copper, oxygen and silver) at the atomic ratio consistent with design compositions of desired Cu_2_O/Ag*x* samples. The molar ratio of Cu to Ag in Cu_2_O/Ag2 and Cu_2_O/Ag5 was 11.3 and 1.25, respectively, decreased with increasing Ag precursor concentration. These results demonstrate that Ag NPs coverage on the surface of Cu_2_O can be tuned by varying the volume of AgNO_3_ and NaBH_4_ solutions. These variations would affect the SERS activity of Cu_2_O/Ag hybrids. Combining the EDS data with the SEM images, we concluded that the Ag NPs were successfully inlaid on Cu_2_O microcubes. Additionally, no other peaks were observed, exhibiting a high purity of the combination. XRD analysis was employed to determine the crystalline phase and structure of Cu_2_O and Cu_2_O/Ag*x* (*x* = 1, 2, 3, 4, 5) (figure 4). All patterns exhibit diffraction peaks centred at 2*θ* = 29°, 36.5°, 42.9°, 63.7° and 77° corresponding to the (110), (111), (200), (220) and (222) crystallographic planes of Cu_2_O cubic phase (JCPDS 05-0667, space group $Pn\bar{3}m$, *a* = 0.4269 nm) [9]. Diffraction peaks at 38.2°, 44.4°, 64.6° and 77.5° are assigned to the (111), (200), (220) and (311) lattice planes of face-centred cubic Ag nanocrystals (JCPDS card no. 04-0783) [5,34,35]. The peak intensity of the (111) plane was relatively high, indicating that the Cu_2_O and Ag crystallites were mainly oriented in this plane [37]. As we can see, the characteristic peaks of Ag have the same position but increase strength with the increase of Ag content. The deposition of Ag NPs did not significantly affect the crystalline structure nature of the Cu_2_O microcubes. No other peaks were seen in these patterns, indicating the high purity of as-prepared samples. On the basis of the XRD patterns, we can conclude that Cu_2_O/Ag*x* was successfully formed with good crystalline property. Figure 5*a* shows the Raman spectra of Cu_2_O and Cu_2_O/Ag*x* samples. When Ag NPs were deposited on Cu_2_O microcubes, the Raman scattering of 290 cm^−1^ peak was notably enhanced, while the Raman scattering of 146 and 215 cm^−1^ peaks was significantly reduced. The intensity of the Raman signal depends strongly on the density of Ag NPs [37]. Figure 5*b* illustrates the FTIR spectra of Cu_2_O and Cu_2_O/Ag*x* with characteristic peaks at 630, 1388 and 1636 cm^−1^ representing the stretching, bending and tensile vibration of Cu-O, confirming the synthesis of Cu_2_O. The broad absorption band at 3498 cm^−1^ is ascribed to the Cu_2_O/Ag bond.

**Figure 6.**
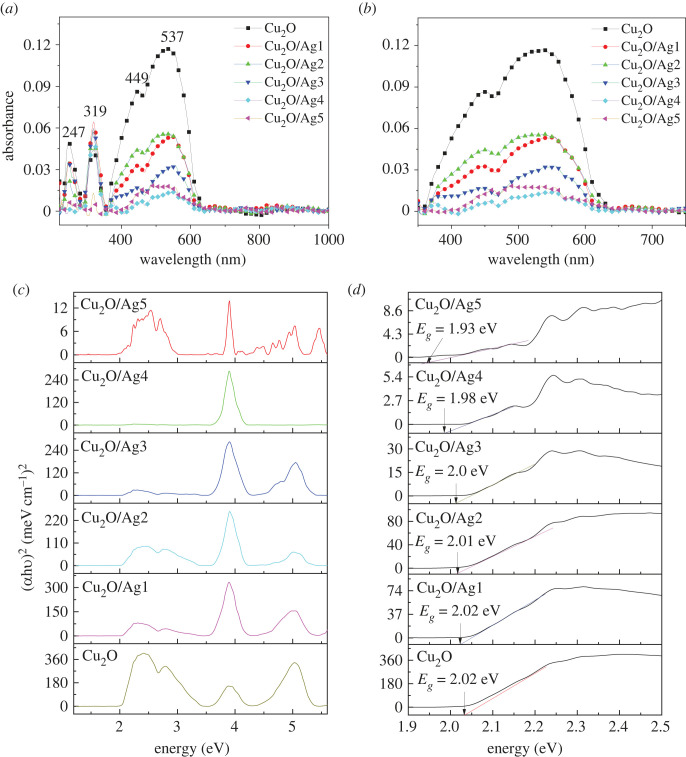
(*a*) UV-Vis-NIR absorption spectra of Cu_2_O and Cu_2_O/Ag assemblies with different Ag amounts in the range of 250–1000 nm, (*b*) expanded view of the 350–750 nm region, (*c*) plots of (*αhν*)^2^ versus photon energy (h*ν*) of Cu_2_O and Cu_2_O/Ag*x* (*x* = 1, 2, 3, 4, 5) and (*d*) energy band gap (*E_g_*) variation with Ag content for all samples.

The optical properties of the materials are of particular interest in addition to morphology specification. Therefore, the UV-Vis absorption spectrum and PL spectrum of Cu_2_O/Ag composite were investigated. The UV-Vis absorption spectroscopy could verify the formation of Cu_2_O/Ag hybrids. Figure 6 presents the UV-Vis-NIR absorption spectra of Cu_2_O and Cu_2_O/Ag with different amounts of Ag NPs. It proves that Cu_2_O/Ag*x* samples with different amounts of Ag NPs have distinguished plasmon absorption characteristics. Cu_2_O and Cu_2_O/Ag*x* show typical absorption peaks around the visible spectrum starting from 350 nm to approximately 650 nm, thus proving the capacity of capturing incident laser irradiation used in this research. Strong absorption peaks were observed at wavelengths shorter than 600 nm and located at 247, 319, 449 and 537 nm. The large, broad peak at 537 nm indicates the formation of Cu_2_O microcrystals [53]. With the introduction of Ag NPs, Cu_2_O/Ag*x* composites exhibit lower absorption of visible light compared with Cu_2_O. The strong absorption peak intensity at 537 nm decreased significantly with the Ag amount increasing, implying that the light absorption in the range from UV to visible is due to Cu_2_O. Thus, the decrease in absorption peak intensity is ascribed to the coverage of Ag NPs on the surface of Cu_2_O microcubes. The surface electrons of Ag made the Cu_2_O microcubes become a polarized surface. UV-Vis spectroscopy was used to explain some features concerning the band gap energy. The band gap energy (*E_g_*) of direct transition semiconductor could be calculated by the experimental Tauc's formula [54],

3.1 $${\left(\alpha h\nu \right)}^{2}=A(h\nu -E_{g}),$$

where *α* presents the absorption coefficient, h is the Planck constant, *ν* is the light frequency, h*ν* exhibits the incident photon energy, *E_g_* is the band gap energy and *A* is the constant. *E_g_* is estimated from the intercept of the tangent of the linear region of (*α*h*ν*)^2^ versus h*ν* curves and energy axis (figure 6*d*).

3.2 $$\alpha =\frac{B}{d},$$

where *B* is the absorbance and *d* is the thickness of the cuvette [55]. The estimated band energies of the samples varying with silver amount are shown in table 2. It is intuitively realized that the addition of Ag NPs narrows the band gap down from 2.02 eV for Cu_2_O to 1.93 eV for Cu_2_O/Ag5. Additionally, according to the UV-Vis spectrum of Cu_2_O/Ag5, a broad peak at 637 nm indicated that the LSPR effect could be excited at approximately 630 nm. Therefore, the 632 nm laser is a proper excitation source for MO detection on the Cu_2_O/Ag SERS substrate.

**Table 2.** The optical band gap energy *E_g_* of Cu_2_O (*x* = 0) and Cu_2_O/Ag*x* (*x* = 1, 2, 3, 4, 5).

*x*	0	1	2	3	4	5
*E_g_* (eV)	2.02	2.02	2.01	2.0	1.98	1.93

**Figure 7.**
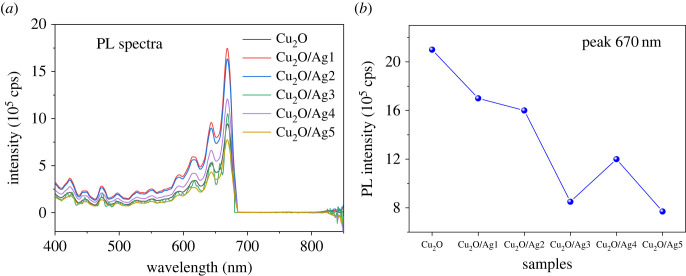
(*a*) The photoluminescence spectra of Cu_2_O and (*b*) PL intensity of 670 nm peak of different samples.

It is well known that the SERS performance of semiconductors is associated with the separation and transfer of photoexcited electron–hole pairs [56,57]. The generation, transport and recombination of photoexcited charge carriers can be investigated by the PL spectroscopy method [58]. The PL peak generally means the recombination of photoinduced electron–hole pairs [59]. A lower PL intensity implies a lower electron–hole recombination rate. Figure 7*a* illustrates the PL spectra of Cu_2_O and Cu_2_O/Ag*x* composites under 325 nm excitation. A distinct narrow peak at 670 nm for all samples is found, which can be indexed to free exciton recombination of Cu_2_O [60]. Cu_2_O microcubes sample shows the highest PL intensity on account of the quickest recombination of charge carriers. Compared with Cu_2_O, Cu_2_O/Ag composites exhibit lower PL intensity, suggesting the incorporation of Ag can effectively suppress the recombination of electron–hole pairs of Cu_2_O. As a result of the introduction of Ag NPs, the lifetime of the charge carriers is prolonged, and the ability to separate and transfer electron–hole pairs is improved [61]. This ability may be due to the Schottky barrier formed at the metal–semiconductor interface, which acts as an electron trap to prevent electron–hole recombination [62]. Thus, the modification with Ag resulted in the introduction of electron traps and acceleration of electron transfer [63].

### 3.2. Surface-enhanced Raman scattering activity toward methyl orange

**Figure 8.**
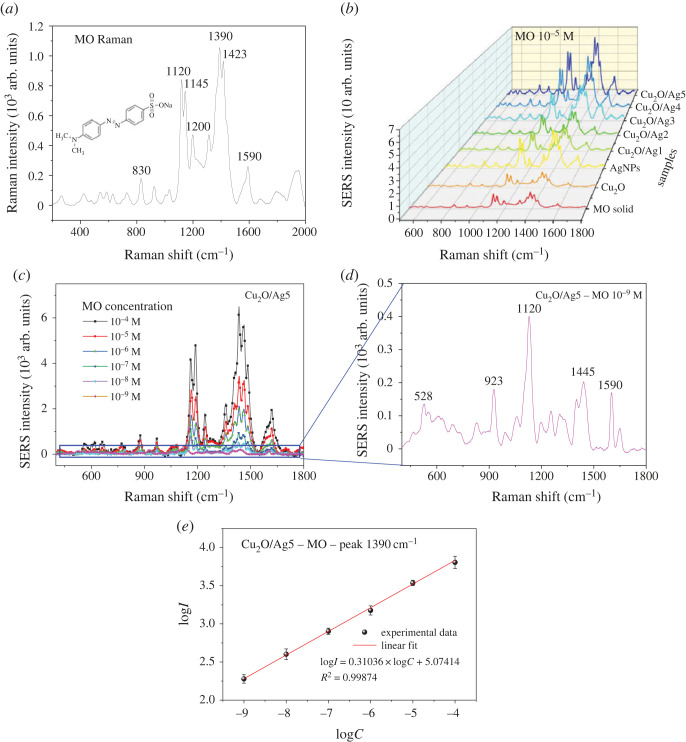
(*a*) Raman spectroscopy of MO, (*b*) SERS spectra of MO (concentration of 10^−5^ M) produced by Cu_2_O and Cu_2_O/Ag*x*, (*c*) SERS spectra of MO with different concentrations in the range of 10^−4^ M and 10^−9^ M produced by Cu_2_O/Ag5, (*d*) SERS spectrum of MO at 10^−9^ M collected by Cu_2_O/Ag5 and (*e*) linear relationship of the logarithm of 1390 cm^−1^ peak intensity versus the logarithm of MO concentration, and the error bars represent the standard deviation.

In recent years, the SERS technique has been widely applied to achieve the trace detection of organic dye molecules with ultrasensitive and non-destructive quantitative analysis results [64,65]. MO was used as a probe molecule to evaluate the SERS activity of Cu_2_O and Cu_2_O/Ag*x* hybrid substrates. The Raman spectra of different substrates were recorded, and the results are illustrated in figure 8. At 532 nm excitation, MO characteristic peaks can be seen in the Raman spectrum in figure 8*a*. In more detail, the peak at 830 cm^−1^ is attributed to the C-N-C skeletal deformation mode; the band at 1120 cm^−1^ is attributed to in-plane ring breathing mode of C-S bond; the peak at 1145 cm^−1^ results from the in-plane bending mode of C-H bond; and the band at 1200 cm^−1^ is related to C-H deformation mode. The peak at 1390 cm^−1^ corresponds to the in-plane aromatic ring deformation mode. The mode at 1423 cm^−1^ results from the N=N bond stretching vibration, and the peak at 1590 cm^−1^ is ascribed to the symmetrical stretching of C-N bonding. It is clear that even at quite high concentration of MO (10^−5^ M), the normal Raman spectrum of MO indicates very low intensity. Cu_2_O slightly enhanced the Raman signal of MO compared with that of the pristine MO. When Cu_2_O/Ag*x* composites are employed as substrates, the strength of the peak at 1390 cm^−1^ is very strong. Many characteristic peaks of MO generated from Cu_2_O/Ag substrates locate at 748, 830, 923, 1120, 1145, 1200, 1390, 1423 and 1590 cm^−1^. The relatively obvious SERS signal of MO appearing at 1390 cm^−1^ is chosen to detect the Raman intensity change. These results indicate that Cu_2_O has a minor improvement on the Raman signal of MO.

The EF and LOD are two essential properties of SERS response. The prior concern of this research is to find out what Cu_2_O/Ag*x* composite will show the highest EF. All samples were implemented under identical experimental conditions. In order to study the synergistic enhancement of Cu_2_O microcubes and Ag nanoparticles, MO (10^−5^ M) was chosen as the probe molecule to explore the Raman spectra of Cu_2_O and Cu_2_O/Ag*x* heterojunctions. Figure 8*b* represents the Raman fingerprint of MO (10^−5^ M) adsorbed on Cu_2_O substrate under blue-green irradiation (532 nm laser). However, after coating Ag NPs on the surface of Cu_2_O, the Raman intensity of MO adsorbed on Cu_2_O/Ag*x* was three–four times greater than that of MO adsorbed on pristine Cu_2_O. The SERS performance of the samples increases as the amount of Ag NPs increases from Cu_2_O/Ag1 to Cu_2_O/Ag5, which indicates that Cu_2_O/Ag5 exhibits the highest amplification. The reason for the different performance originated from Cu_2_O/Ag heterostructural feature. The intense coupling of Ag NPs and the incident laser enabled prominent CT from Ag NPs to the MO molecules. The Cu_2_O/Ag5 substrate with the best Raman response was selected for performing SERS probing as a function of MO concentration. The SERS intensity increased with increasing MO concentration in the Cu_2_O/Ag5 system (figure 8*c*). Cu_2_O/Ag5 presented a distinguishable SERS signal at a concentration of MO down to 10^−9^ M, as exhibited in figure 8*d*. We chose 1 nM to be the LOD of Cu_2_O/Ag5 SERS substrate toward MO. These results verify that Cu_2_O/Ag5 has a high sensitivity for detecting MO, which is suitable for the low-concentration detection of the specific material.

For the Cu_2_O/Ag5 sample, the logarithm of the Raman intensity of 1390 cm^−1^ peak obeys the linear dependence relationship with the logarithm of MO concentration among $10^{-9}$–10^−4^ M and the corresponding regression equation is log *I* = 0.31036log *C* + 5.07414, and the linear correlation coefficient (*R*^2^) was 0.99874 (figure 8*e*). In order to verify the stability of Cu_2_O/Ag5, we compared SERS spectra from the same MO concentration at different time intervals (1, 7 and 30 days) and observed no obvious change in Raman spectra. Therefore, Cu_2_O/Ag5 is stable as a SERS substrate to detect MO. In order to test whether Cu_2_O/Ag5 is recyclable in practical applications, three cycles were performed by successive SERS measurement and rinsing by alcohol. The position of characteristic peaks in each cycle is similar, but the following peak intensity is slightly reduced (about 95% of the initial strength, data not shown here).

**Figure 9.**
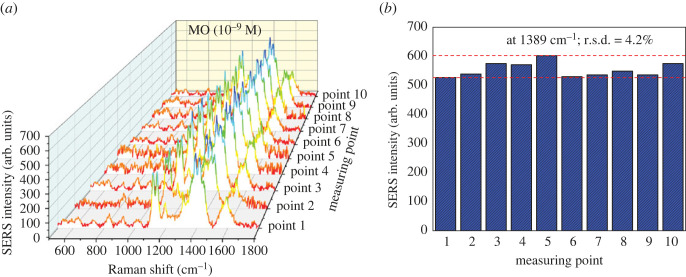
(*a*) SERS spectra of 10^−9^ M MO collected by Cu_2_O/Ag5 from 10 random spots, (*b*) the SERS intensities of 1390 cm^−1^ peak from 10 random points.

To evaluate the uniformity of the SERS substrates, SERS spectra of MO (10^−9^ M) on Cu_2_O/Ag5 were received from 10 random points (figure 9*a*). The spatial distribution of SERS intensity at 1390 cm^−1^ is shown in figure 9*b*. The relative standard deviation (r.s.d.) of the Raman intensity of these spots was calculated to be 4.2%, which confirms the uniformity of Cu_2_O/Ag5 substrate.

In addition, the SERS EF of the Cu_2_O/Ag5 substrate was estimated according to the following equation [15]:

3.3 $$EF=\frac{I_{\mathrm{SERS}}}{I_{o}}\cdot \frac{N_{o}}{N_{\mathrm{SERS}}},$$

where *I*_SERS_ is the SERS intensity of MO solution after adsorption on the Cu_2_O/Ag5 substrate, *I*_o_ is the Raman intensity of MO powder on the glass substrate, and *N*_SERS_ and *N*_o_ are the corresponding numbers of MO molecules in the laser spot.

3.4 $$N_{o}=\rho hS\frac{N_{A}}{M},$$

where *S* is the laser spot diameter of 1 µm, *ρ* is the density of the MO molecules of 1.28 g cm^−3^ [66], h is the effective laser depth of 19 µm, *N_A_* = 6.023 × 10^23^ mol^−1^ is the Avogadro constant, *M* is the molar mass of MO (327.33 g mol^−1^). It is assumed that MO molecules were adsorbed as a monolayer on the surface of Cu_2_O/Ag5 substrate, *N*_SERS_ is determined in the illuminated volume of Raman set-up as follows [34]:

3.5 $$N_{\mathrm{SERS}}=\frac{S}{S_{\mathrm{MO}}},$$

where *S*_MO_ is the surface area of a MO molecule (approx. 0.17 nm^2^). The EF values for the Raman peaks of MO located at 923, 1120, 1145, 1200, 1390, 1423 and 1590 cm^−1^ were estimated and summarized in table 3. The EF acquires for 10^−5^ MO of MO giving the following values: 144.6 × 10^3^ for 923 cm^−1^ peak, 684.6 × 10^3^ for 1120 cm^−1^ peak, 632.4 × 10^3^ for 1145 cm^−1^ peak, 304 × 10^3^ for 1200 cm^−1^ peak, 1.112 × 10^6^ for 1390 cm^−1^ peak, 846 × 10^3^ for 1423 cm^−1^ and 284 × 10^3^ for 1590 cm^−1^, respectively. As can be seen, the most intensive peak of MO is located at 1390 cm^−1^. The linear range, LOD and EF of various substrates in detecting MO are summarized in table 4. The Cu_2_O microcubes/Ag nanoparticles structure exhibits one order of magnitude higher EF than that of C/Ag, Si/Ag or pristine Ag. EF of Cu_2_O/Ag5 in this work reaches the highest value among SERS substrates reported in literature. Those platforms failed to detect MO at a concentration lower than 10^−9^ M, which is not the case in the current research, where MO fingerprint was well defined at 10^−9^ M. In addition, Cu_2_O/Ag demonstrates at least one order of magnitude wider linear range compared with those reported elsewhere [48,49].

**Table 3.** The EF was calculated from various SERS peaks of Cu_2_O/Ag5 substrate with the MO concentration in the range of $10^{-4}\;$M to $10^{-9}\;$M.

	EF						
C (M)	$923\;{\mathrm{cm}}^{-1}$	$1120\;{\mathrm{cm}}^{-1}$	$1145\;{\mathrm{cm}}^{-1}$	$1200\;{\mathrm{cm}}^{-1}$	$1390\;{\mathrm{cm}}^{-1}$	$1423\;{\mathrm{cm}}^{-1}$	$1590\;{\mathrm{cm}}^{-1}$
$10^{-4}$	$17.32\times 10^{3}$	$84.08\times 10^{3}$	$97.8\times 10^{3}$	$35.26\times 10^{3}$	$130\times 10^{3}$	$113\times 10^{3}$	$39.45\times 10^{3}$
$10^{-5}$	$144.6\times 10^{3}$	$684.6\times 10^{3}$	$632.4\times 10^{3}$	$304\times 10^{3}$	$1.112\times 10^{6}$	$846\times 10^{3}$	$284\times 10^{3}$
$10^{-6}$	$0.72\times 10^{6}$	$3.462\times 10^{6}$	$2.812\times 10^{6}$	$1.414\times 10^{6}$	$4.212\times 10^{6}$	$3.962\times 10^{6}$	$1.316\times 10^{6}$
$10^{-7}$	$3.56\times 10^{6}$	$22.3\times 10^{6}$	$14.66\times 10^{6}$	$6.2\times 10^{6}$	$19.04\times 10^{6}$	$16.94\times 10^{6}$	$5.74\times 10^{6}$
$10^{-8}$	$21\times 10^{6}$	$172\times 10^{6}$	$78\times 10^{6}$	$38.8\times 10^{6}$	$98\times 10^{6}$	$95.2\times 10^{6}$	$33.8\times 10^{6}$
$10^{-9}$	$202\times 10^{6}$	$152\times 10^{6}$	$1.03\times 10^{9}$	$206\times 10^{6}$	$388\times 10^{6}$	$444\times 10^{6}$	$550\times 10^{6}$

**Table 4.** Comparison of various SERS substrates for the detection of MO.

materials	linear range	LOD	EF	reference
C/Ag	(6–9) × 10^−3^ M	$6.11\times 10^{-3}\;M$	$2.91\times 10^{8}$	[49]
Si/Ag	$10^{-9}$–10^−3^ M	$10^{-9}\;M$	not given	[48]
Ag	not given	$5\times 10^{-7}\;M$	$8.9\times 10^{6}$	[67]
Cu_2_O/Ag	$10^{-9}$–10^−4^ M	$10^{-9}\;M$	$3.88\times 10^{8}$	this work

### 3.3. The mechanism of surface-enhanced Raman scattering-based analysis

Cu_2_O/Ag*x* (*x* = 1–4) show less enhancement in the Raman signal, and the Raman signal of Cu_2_O/Ag1 substrate with the lowest Ag loading is the weakest. These results could probably be ascribed to few dispersed Ag NPs resulting in fewer hot spots and the subtle LSPR effect. It is supposed that the surface area of Ag NPs had a tendency to increase as the amount of Ag increased, which may have been the reason for the increase in the SERS intensity for the Cu_2_O/Ag5 sample. For Cu_2_O/Ag5 substrate, the SERS activity is strengthened, owing to a large number of hot spots generated between the Cu_2_O microcubes and Ag nanoparticles [34]. Moreover, Cu_2_O/Ag5 owns a rougher surface than Cu_2_O and Cu_2_O/Ag*x* (*x* = 1–4), thus resulting in many more interparticle Raman hot spots. The enhancement of the electromagnetic field of Cu_2_O/Ag*x* compared with Cu_2_O is related to the hot spot [37]. The gaps between Cu_2_O microcubes are too wide, as a result, the optically induced electromagnetic field is insignificantly generated by separated Cu_2_O, giving a low enhancement. In the case of Cu_2_O/Ag composites, the interparticle distances are significantly shorter, which provides a highly intense electromagnetic field to enhance the SERS signals significantly [5,38]. Additionally, a clear increase of SERS signal is observed by increasing the content of Ag NPs from Cu_2_O to Cu_2_O/Ag5, indicating that Cu_2_O/Ag5 possesses maximal SERS intensity correlative with the maximal quantity of hot spots. This finding proves that Cu_2_O/Ag5 heterostructure is more beneficial for exploitation as a SERS substrate in detecting MO molecules.

When the laser was irradiated on the rough substrate, the electromagnetic wave excited the free electrons in the substrate to produce a collective oscillation called a surface plasmon. When the incident light frequency matches the surface plasmon frequency, LSPR is generated, which greatly increases the local electric field near the surface, leading to the Raman signal enhancement. Another possibility for SERS enhancement is the quantum confinement between the LSPR of Ag NPs and the cavity of the Cu_2_O microcubes [68].

The separation efficiency of photoexcited charges of Cu_2_O was improved significantly after Ag NPs covered the Cu_2_O surfaces due to a Schottky barrier at the metal–semiconductor interface. The heterojunction formed between Cu_2_O and Ag promoted the transfer of electrons and holes to the surface of Ag and Cu_2_O [69].

**Figure 10.**
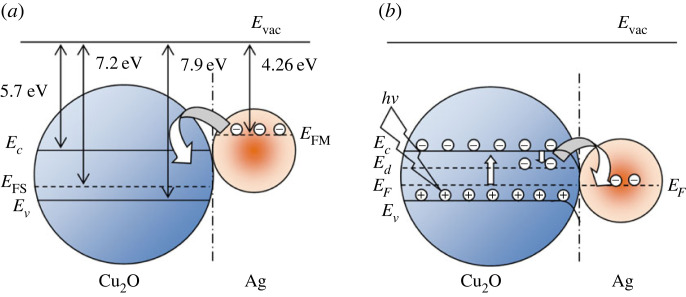
(*a*) Schematic of an inner electric field formation process between Ag and Cu_2_O and (*b*) the charge separation and transfer process after laser irradiation.

The dominated CT from Ag to Cu_2_O leads to the high density of electrons on the surface of Cu_2_O microcubes, promoting the charge penetration between Cu_2_O and the analyte. The laser excitation and the oscillating electrons would excite the analyte, resulting in the probe molecules' polarization. As a result, the scattered light is much stronger than normal Raman scattering [5]. The conduction band (CB) and valence band (VB) of pure Cu_2_O were 5.7 and 7.9 eV from the vacuum level, respectively [9]. In Cu_2_O/Ag heterostructure, the Fermi level of Ag is 4.26 eV from the vacuum level [70], higher than that of Cu_2_O (7.2 eV from the vacuum level, 0.5 eV above VB) [71]. A Schottky junction can be built at the interface of the semiconductor (Cu_2_O) and the metal (Ag), so the electrons transfer from Ag to the CB of Cu_2_O until the energy thermodynamic equilibrium level [72]. The Fermi level of Cu_2_O (*E*_FS_) will be raised, and the Fermi level of Ag (*E*_FM_) will be lowered, thus curving the energy band at the Cu_2_O/Ag interface. The CB edge of Cu_2_O is higher than the Fermi level of the thermodynamic equilibrium state (figure 10*b*).

The Raman excitation wavelength at 532 nm corresponding to the excitation energy of a light photon of 2.33 eV, higher than the band gap (2.17 eV) of the Cu_2_O, generates the photoinduced electron–hole pairs. The electrons in the CB of Cu_2_O migrate to Ag NPs. However, the electrons in the Ag NPs are not able to transfer to Cu_2_O due to the Schottky barrier. Consequently, the photoexcited electron–hole pairs can be effectively separated. The energy gap (1.71 eV) between the MO molecule's lowest unoccupied molecular orbital (LUMO) and highest occupied molecular orbital (HOMO) [73] facilitates the CT effect of the substrate and the targeted molecules. Ag NPs absorb 532 nm light to generate LSPR and excite a strong electric field. The synergistic effect between the heterostructure and the homogeneous dispersion of hot spots formed by Ag NPs contributed to Raman intensity [69]. Furthermore, the direct contact and CT between Cu_2_O and MO molecules play a role in signal enhancement. MO molecules were adsorbed on the surface of Cu_2_O/Ag*x* via chemical bonds, and then, electrons are transferred from metal to MO molecules, resulting in the local change in the electron density near the surface. This phenomenon leads to the chemical enhancement of the SERS signal [49]. In addition, a defect level (*E*_d_) formed near the bottom of the Cu_2_O CB can accelerate the CT of the Cu_2_O/Ag sample. Due to the LSPR effect of the noble metal Ag, the photoexcited electrons were injected into the CB of Cu_2_O, and vibrational relaxed to the defect level (*E*_d_) in Cu_2_O. The CB and E_d_ of Cu_2_O served as a bridge between Ag NPs (donor) and MO molecules (acceptor) [74].

## 4. Conclusion

In conclusion, we successfully synthesized Cu_2_O microcubes via solvothermal method and coated Ag nanoparticles on their surfaces through reduction reaction. The coverage density of Ag NPs on the surface of Cu_2_O microcubes could be tuned by varying the amount of silver precursor as well as a reduction agent. XRD, SEM, EDS, UV-Vis, Raman and FTIR results confirmed that Ag NPs were attached to the surface of Cu_2_O microcubes. Cu_2_O microcubes (0.5–1 µm) were decorated by Ag NPs (15–20 nm). The *E_g_* value of Cu_2_O/Ag*x* composites was calculated from UV-Vis measurements. The results suggested that the addition of Ag NPs narrowed down the band gap, which was helpful for the adsorption in the visible light region. In addition, the SERS activity of Cu_2_O/Ag systems was evaluated in detecting MO. The optimal SERS performance in Cu_2_O/Ag*x* series was Cu_2_O/Ag5 with both high sensitivity and good producibility advances. At 532 nm excitation, the Cu_2_O/Ag5 hybrid showed excellent SERS activity of MO analyte with an EF reaching 3.88 × 10^8^, a detection limit of 10^−9^ M, and an r.s.d. of 4.2%. The excellent SERS performance may be due to the CT between Ag and Cu_2_O and a large number of hot spots at the metal–semiconductor interface. Compared with pristine Cu_2_O microcubes, Cu_2_O/Ag5 exhibited outstanding SERS enhancement, because the modification with Ag NPs introduced more electron traps and accelerated the electron transfer between the Cu_2_O and MO molecules. The logarithm of SERS intensity shows a linear relationship with the logarithm of MO concentration in the range from 1 nM to 0.1 mM. Additionally, the surface area of Ag NPs is an important factor influencing the SERS intensity. The greater the surface area of silver (larger amount of silver), the greater the SERS signal. Cu_2_O/Ag5 exhibited a sensitive, rapid and low-cost SERS substrate with great potential for detecting organic pollutants for food safety testing, environmental monitoring and other industrial fields. These findings in this study will be of great significance for developing SERS platforms for detecting the targeted molecules and discovering water pollution, especially the MO detection device driven by visible light.

## Data Availability

This article does not contain any additional data.

## References

[1] Fleischmann M, Hendra PJ, McQuillan AJ. 1974 Raman spectra of pyridine adsorbed at a silver electrode. Chem. Phys. Lett. **26**, 163-166. (10.1016/0009-2614(74)85388-1)

[2] Shen Z, Wang H, Yu Q, Li Q, Lu X, Kong X. 2021 On-site separation and identifiation of polycyclic aromatic hydrocarbons from edible oil by TLC-SERS on diatomite photonic biosilica plate. Microchem. J. **160**, 105672. (10.1016/j.microc.2020.105672)

[3] Nie X, Chen Z, Tian Y, Chen S, Que L, Fan M. 2021 Rapid detection of trace formaldehyde in food based on surface-enhanced Raman scattering coupled with assembled purge trap. Food Chem. **340**, 127930. (10.1016/j.foodchem.2020.127930) 32871357

[4] Chen Q, Shi C, Qin L, Kang S-Z, Li X. 2021 A low-cost 3D core-shell nanocomposite as ultrasensitive and stable surface enhanced Raman spectroscopy substrate. Sens. Actuators B **327**, 128907. (10.1016/j.snb.2020.128907)

[5] Li Y, Wang Y, Wang M, Zhang J, Wang Q, Li H. 2020 A molecularly imprinted nanoprobe incorporating Cu_2_O@Ag nanoparticles with different morphologies for selective SERS based detection of chlorophenols. Microchim. Acta **187**, 59. (10.1007/s00604-019-4052-y) 31848711

[6] Vu XH, Dien ND, Pham TT, Van Truong N, Ca NX, Van Thu V. 2021 Tunable LSPR of silver/gold bimetallic nanoframes and their SERS activity for methyl red detection. RSC Adv. **11**, 14 596-14 606. (10.1039/D1RA01477C) PMC8698163 35423967

[7] De Paepe E, Wauters J, Van Der Borght M, Claes J, Huysman S, Croubels S, Vanhaecke L. 2019 Ultra-high-performance liquid chromatography coupled to quadrupole orbitrap high-resolution mass spectrometry for multi-residue screening of pesticides, (veterinary) drugs and mycotoxins in edible insects. Food Chem. **293**, 187-196. (10.1016/j.foodchem.2019.04.082) 31151600

[8] Paloglou A, Martakidis K, Gavril D. 2017 An inverse gas chromatographic methodology for studying gas-liquid mass transfer. J. Chromatogr. A **1480**, 83-92. (10.1016/j.chroma.2016.12.034) 27993392

[9] Guo L, Mao Z, Jin S, Zhu L, Zhao J, Zhao B, Jung YM. 2021 A SERS study of charge transfer process in Au nanorod–MBA@Cu_2_O assemblies: effect of length to diameter ratio of Au nanorods. Nanomaterials **11**, 867. 33805298 10.3390/nano11040867 PMC8066000

[10] Ouyang H, Liang A, Jiang Z. 2018 A simple and selective resonance Rayleigh scattering-energy transfer spectral method for determination of trace neomycin sulfate using Cu_2_O particle as probe. Spectrochim. Acta Part A **190**, 268-273. (10.1016/j.saa.2017.09.018) 28946076

[11] Ai YJ, Liang P, Wu YX, Dong QM, Li JB, Bai Y, Xu BJ, Yu Z, Ni D. 2018 Rapid qualitative and quantitative determination of food colorants by both Raman spectra and surface-enhanced Raman scattering (SERS). Food Chem. **241**, 427-433. (10.1016/j.foodchem.2017.09.019) 28958550

[12] Chen L et al. 2016 Design of Cu_2_O-Au composite microstructures for surface-enhanced Raman scattering study. Colloids Surf. A **507**, 96-102. (10.1016/j.colsurfa.2016.07.053)

[13] Prinz J, Heck C, Ellerik L, Merk V, Bald I. 2016 DNA origami based Au–Ag-core–shell nanoparticle dimers with single-molecule SERS sensitivity. Nanoscale **8**, 5612-5620. (10.1039/C5NR08674D) 26892770 PMC4778414

[14] Jiao A, Zhang H, Xu L, Tian Y, Liu X, Chen M, Chen F. 2019 Core-shell Au@Ag nanodendrites supported on TiO_2_ nanowires for blue laser beam-excited SERS-based pH sensing. Opt. Express **27**, 23 981-23995. (10.1364/OE.27.023981) 31510294

[15] Huang J, Zhou T, Zheng H, Wang J, Jiang Y, Zhang Y, Liu Y. 2022 Construction of ternary multifunctional Fe3O4/Cu2O/Au nanocomposites: SERS detection and visible light driven photocatalysis for organic dyes. Ceram. Int. **48**, 25 413-25 423. (10.2139/ssrn.4001381)

[16] Jiao A, Xu L, Tian Y, Cui Q, Liu X, Chen M. 2021 Cu_2_O nanocubes–grafted highly dense Au nanoparticles with modulated electronic structures for improving peroxidase catalytic performances. Talanta **225**, 121990. (10.1016/j.talanta.2020.121990) 33592738

[17] Liu M, Xiang R, Lee Y, Otsuka K, Ho Y-L, Inoue T, Chiashi S, Delaunay JJ, Maruyama S. 2018 Fabrication, characterization, and high temperature surface enhanced Raman spectroscopic performance of SiO_2_ coated silver particles. Nanoscale **10**, 5449-5456. (10.1039/C7NR08631H) 29493702

[18] Barbillon G. 2019 Fabrication and SERS performances of metal/Si and metal/ZnO nanosensors: a review. Coat. **9**, 86.

[19] Liu B, Zhang W, HaoMing Lv DZ, Gong X. 2012 Novel Ag decorated biomorphic SnO_2_ inspired by natural 3D nanostructures as SERS substrates. Mater. Lett. **74**, 43-45. (10.1016/j.matlet.2011.12.086)

[20] Yang Q, Wang J, Wu H, Qin S, Pan J, Li C. 2022 Hierarchically rough CuO/Ag composite film with controlled morphology as recyclable SERS-active substrate. Appl. Surf. Sci. **598**, 153746. (10.1016/j.apsusc.2022.153746)

[21] Yao C, Gao X, Liu X, Shen Y, Xie A. 2021 In-situ preparation of Ferrero® chocolate-like Cu_2_O@Ag microsphere as SERS substrate for detection of thiram. J. Mater. Res. Technol. **11**, 857-865. (10.1016/j.jmrt.2021.01.069)

[22] Gao W et al. 2020 Electromagnetic induction derived micro-electric potential in metal-semiconductor core-shell hybrid nanostructure enhancing charge separation for high performance photocatalysis. Nano Energy **71**, 104624. (10.1016/j.nanoen.2020.104624)

[23] Vu XH, Dien ND, Pham TT, Trang TT, Ca NX, Tho PT, Vinh ND, Van Do P. 2020 The sensitive detection of methylene blue using silver nanodecahedra prepared through a photochemical route. RSC Adv. **10**, 38 974-38 988. (10.1039/D0RA07869G) PMC9057378 35518425

[24] Li Q, Gong S, Zhang H, Huang F, Zhang L, Li S. 2019 Tailored necklace-like Ag@ZIF-8 core/shell heterostructure nanowires for high-performance plasmonic SERS detection. Chem. Eng. J. **371**, 26-33. (10.1016/j.cej.2019.03.236)

[25] Gao H, Zhang J, Li M, Liu K, Guo D, Zhang Y. 2013 Evaluating the electric property of different crystal faces and enhancing the Raman scattering of Cu_2_O microcrystal by depositing Ag on the surface. Curr. Appl. Phys. **13**, 935-939. (10.1016/j.cap.2013.01.049)

[26] Xu JQ, Duo HH, Zhang YG, Zhang XW, Fang W, Liu YL, Shen AG, Hu JM, Huang WH. 2016 Photochemical synthesis of shape-controlled nanostructured gold on zinc oxide nanorods as photocatalytically renewable sensors. Anal. Chem. **88**, 3789-3795. (10.1021/acs.analchem.5b04810) 26928162

[27] He X, Yue C, Zang Y, Yin J, Sun S, Li J, Kang J. 2013 Multi-hot spot configuration on urchin-like Ag nanoparticle/ZnO hollow nanosphere arrays for highly sensitive SERS. J. Mater. Chem. A **1**, 15 010-15 015. (10.1039/C3TA13450D)

[28] Ribbing CG, Roos A. 1991 Copper oxides (Cu_2_O, CuO). In Handbook of optical constants of solids (ed. ED Palik), pp. 875-882. San Diego, CA: Academic Press. (10.1016/B978-012544415-6.50085-6)

[29] Dumitru C, Muscurel VF, Fara L. 2018 Cu_2_O layer analysis and optimization based on a metal-oxide tandem heterojunction solar cell. Mater. Today Proc. **5**, 15 895-15 901. (10.1016/j.matpr.2018.06.060)

[30] Muthulakshmi L, Rajini N, Nellaiah H, Kathiresan T, Jawaid M, Varada Rajulu A. 2017 Preparation and properties of cellulose nanocomposite films with *in situ* generated copper nanoparticles using *Terminalia catappa* leaf extract. Int. J. Biol. Macromol. **95**, 1064-1071. (10.1016/j.ijbiomac.2016.09.114) 27984140

[31] Sakar M, Balakumar S. 2018 Reverse Ostwald ripening process induced dispersion of Cu_2_O nanoparticles in silver-matrix and their interfacial mechanism mediated sunlight driven photocatalytic properties. J. Photochem. Photobiol. A **356**, 150-158. (10.1016/j.jphotochem.2017.12.040)

[32] Wang M, Zhang S, Li M, Han A, Zhu X, Ge Q, Han J, Wang H. 2020 Facile synthesis of hierarchical flower-like Ag/Cu_2_O and Au/Cu_2_O nanostructures and enhanced catalytic performance in electrochemical reduction of CO_2_. Front. Chem. Sci. Eng. **14**, 813-823. (10.1007/s11705-019-1854-8)

[33] Zhao Y, Liu H, Shi L, Zheng W, Jing X. 2020 Electroactive Cu_2_O nanoparticles and Ag nanoparticles driven ratiometric electrochemical aptasensor for prostate specific antigen detection. Sens. Actuators B **315**, 128155. (10.1016/j.snb.2020.128155)

[34] Wu T et al. 2021 Self-sustainable and recyclable ternary Au@Cu_2_O–Ag nanocomposites: application in ultrasensitive SERS detection and highly efficient photocatalysis of organic dyes under visible light. Microsyst. Nanoeng. **7**, 23. (10.1038/s41378-021-00250-5) 34567737 PMC8433429

[35] Hong D, Cao G, Qu J, Deng Y, Tang J. 2018 Antibacterial activity of Cu_2_O and Ag co-modified rice grains-like ZnO nanocomposites. J. Mater. Sci. Technol. **34**, 2359-2367. (10.1016/j.jmst.2018.06.011)

[36] Praveen R, Ramaraj R. 2019 Facile synthesis of hetero-nanostructured cuprous oxide-gold composite material for sensitive enzymeless glucose detection. J. Electroanal. Chem. **851**, 113454. (10.1016/j.jelechem.2019.113454)

[37] Luo Y, Xing L, Hu C, Zhang W, Lin X, Gu J. 2022 Facile synthesis of nanocellulose-based Cu_2_O/Ag heterostructure as a surface-enhanced Raman scattering substrate for trace dye detection. Int. J. Biol. Macromol. **205**, 366-375. (10.1016/j.ijbiomac.2022.02.102) 35192906

[38] Jiao A et al. 2022 Double profound enhancements of Cu_2_O nano-octahedrons connected by intertwined Ag nanovines for elevating SERS activity toward ultrasensitive pesticide detection. Opt. Express **30**, 586-602. (10.1364/OE.444937) 35201233

[39] Lin J, Hao W, Shang Y, Wang X, Qiu D, Ma G, Chen C, Li S, Guo L. 2017 Direct experimental observation of facet-dependent SERS of Cu_2_O polyhedra. Small **14**, 1703274. (10.1002/smll.201703274) 29239098

[40] Wang RC, Lin HY. 2012 Efficient surface enhanced Raman scattering from Cu_2_O porous nanowires transformed from CuO nanowires by plasma treatments. Mater. Chem. Phys. **136**, 661-665. (10.1016/j.matchemphys.2012.07.039)

[41] Liu S, Kang M, Yan F, Peng D, Yang Y, He L, Wang M, Fang S, Zhang Z. 2015 Electrochemical DNA biosensor based on microspheres of cuprous oxide and nano-chitosan for Hg(II) detection. Electrochim. Acta **160**, 64-73. (10.1016/j.electacta.2015.02.030)

[42] Yang Z, Ma C, Wang W, Zhang M, Hao X, Chen S. 2019 Fabrication of Cu_2_O-Ag nanocomposites with enhanced durability and bactericidal activity. J. Colloid Interface Sci. **557**, 156-167. (10.1016/j.jcis.2019.09.015) 31520996

[43] Shvalya V, Filipič G, Vengust D, Zavašnik J, Modic M, Abdulhalim I, Cvelbar U. 2020 Reusable Au/Pd-coated chestnut-like copper oxide SERS substrates with ultra-fast self-recovery. Appl. Surf. Sci. **517**, 146205. (10.1016/j.apsusc.2020.146205)

[44] Liu T, Liu Q, Hong R, Tao C, Wang Q, Lin H, Han Z, Zhang D. 2021 Cuprous oxide induced the surface enhanced Raman scattering of silver thin fims. Chem. Phys. Lett. **783**, 139071. (10.1016/j.cplett.2021.139071)

[45] Luo H, Zhou J, Zhong H, Zhou L, Jia Z, Tan X. 2016 Polyhedron Cu_2_O@Ag composite microstructures: synthesis, mechanism analysis and structure-dependent SERS properties. RSC Adv. **6**, 99 105-99 113. (10.1039/C6RA20856H)

[46] Velusamy K, Periyasamy S, Kumar PS, Femina Carolin C, Jayaraj T, Gokulakrishnan M, Keerthana P. 2022 Transformation of aqueous methyl orange to green metabolites using bacterial strains isolated from textile industry effluent. Environ. Technol. Innov. **25**, 102126. (10.1016/j.eti.2021.102126)

[47] Bashir I, Lone FA, Bhat RA, Mir SA, Dar ZA, Dar SA. 2020 Concerns and threats of contamination on aquatic ecosystems. In Bioremediation and biotechnology (ed. KR Hakeem), pp. 1-26. Cham, Switzerland: Springer Nature. (doi:10.1007/978-3-030-35691-0_1).

[48] Van Nguyen T, Vu DC, Pham VH, Pham TB, Pham VH, Bui H. 2021 Improvement of SERS for detection of ultra-low concentration of methyl orange by nanostructured silicon decorated with Ag nanoparticles. Opt. Int. J. Light Electron Opt. **231**, 166431. (10.1016/j.ijleo.2021.166431)

[49] Zarei A, Shafikhani A. 2020 Surface-enhanced Raman scattering (SERS) of methyl orange on Ag-DLC nanoparticles. Mater. Chem. Phys. **242**, 122559. (10.1016/j.matchemphys.2019.122559)

[50] Si MZ, Kang YP, Zhang ZG. 2009 Surface-enhanced Raman scattering (SERS) spectra of methyl orange in Ag colloids prepared by electrolysis method. Appl. Surf. Sci. **255**, 6007-6010. (10.1016/j.apsusc.2009.01.055)

[51] Scaramuzza S, Polizzi S, Amendola V. 2019 Magnetic tuning of SERS hot spots in polymer-coated magnetic–plasmonic iron–silver nanoparticles. Nanoscale Adv. **1**, 2681-2689. (10.1039/C9NA00143C) 36132716 PMC9417711

[52] Shafi M, Liu R, Zha Z, Li C, Du X, Wali S, Jiang S, Man B, Liu M. 2021 Highly efficient SERS substrates with different Ag interparticle nanogaps based on hyperbolic metamaterials. Appl. Surf. Sci. **555**, 149729. (10.1016/j.apsusc.2021.149729)

[53] Sharma K, Maiti K, Kim NH, Hui D, Lee JH. 2018 Green synthesis of glucose-reduced graphene oxide supported Ag-Cu_2_O nanocomposites for the enhanced visible-light photocatalytic activity. Compos. B **138**, 35-44. (10.1016/j.compositesb.2017.11.021)

[54] Feng T, Zhao K, Li H, Wang W, Dong B, Cao L. 2021 Constructing a 2D/2D heterojunction of MoSe_2_/ZnIn_2_S_4_ nanosheets for enhanced photocatalytic hydrogen evolution. Cryst. Eng. Comm. **23**, 2547-2555. (10.1039/D0CE01808B)

[55] Abdallah FB, Benali A, Triki M, Dhahri E, Nomenyo K, Lerondel G. 2019 Investigation of structural, morphological, optical and electrical properties of double-doping lanthanum ferrite. J. Mater. Sci. **30**, 3349-3358. (10.1007/s10854-018-00608-y)

[56] Kumar EA, Wang TJ, Chang YH. 2022 Ultrasensitive SERS substrates based on Au nanoparticles photo-decorated on Cu_2_O microspheres for the detection of rhodamine B and methylene blue. Appl. Surf. Sci. **585**, 152696. (10.1016/j.apsusc.2022.152696)

[57] Lin J, Shang Y, Li X, Yu J, Wang X, Guo L. 2017 Ultrasensitive SERS detection by defect engineering on single Cu_2_O superstructure particle. Adv. Mater. **29**, 1604797. (10.1002/adma.201604797) 27892634

[58] Yang S, Yao J, Quan Y, Hu M, Su R, Gao M, Han D, Yang J. 2020 Monitoring the charge-transfer process in a Nd-doped semiconductor based on photoluminescence and SERS technology. Light **9**, 117. (10.1038/s41377-020-00361-0) PMC7351777 32685138

[59] Hou X, Cui L, Du H, Gu L, Li Z, Yuan Y. 2020 Lowering the Schottky barrier of g-C_3_N_4_/carbon graphite heterostructure by N-doping for increased photocatalytic hydrogen generation. Appl. Catal. B **278**, 119253. (10.1016/j.apcatb.2020.119253)

[60] Shao Z, Zhang Y, Yang X, Zhong M. 2020 Au-mediated charge transfer process of ternary Cu_2_O/Au/TiO_2_-NAs nanoheterostructures for improved photoelectrochemical performance. ACS Omega **5**, 7503-7518. (10.1021/acsomega.0c00299) PMC7144151 32280894

[61] Fu S, Chen J, Han H, Wang W, Shi H, Fu J, Jia Y. 2019 ZnO@Au@Cu_2_O nanotube arrays as efficient visible-light-driven photoelectrod. J. Alloy. Compd. **799**, 183-192. (10.1016/j.jallcom.2019.05.340)

[62] Wei Q et al. 2018 Construction of rGO wrapping octahedral Ag-Cu_2_O heterostructure for enhanced visible light photocatalytic activity. Appl. Catal. B Environ. **227**, 132-144. (10.1016/j.apcatb.2018.01.003)

[63] Li L, Zheng X, Chi Y, Wang Y, Sun X, Yue Q, Gao B, Xu S. 2020 Molecularly imprinted carbon nanosheets supported TiO_2_: strong selectivity and synergic adsorption-photocatalysis for antibiotics removal. J. Hazard. Mater. **383**, 121211. (10.1016/j.jhazmat.2019.121211) 31546219

[64] Liu Y et al. 2018 Detection and identification of estrogen based on surface-enhanced resonance Raman scattering (SERRS). Mol. **23**, 1330. (10.3390/molecules23061330) PMC6099535 29857591

[65] Han D, Li B, Chen Y, Wu T, Kou Y, Xue X, Chen L, Liu Y, Duan Q. 2019 Facile synthesis of Fe_3_O_4_@Au core–shell nanocomposite as a recyclable magnetic surface enhanced Raman scattering substrate for thiram detection. Nanotechnology **30**, 465703. (10.1088/1361-6528/ab3a84) 31476137

[66] Sandberg RG, Henderson GH, White RD, Eyring EM. 1972 Kinetics of acid dissociation-ion recombination of aqueous methyl orange. J. Phys. Chem. **76**, 4023-4025. (10.1021/j100670a024)

[67] Liu J, Zhang C, Zhang S, Yu H, Xie W. 2020 A versatile β-cyclodextrin functionalized silver nanoparticle monolayer for capture of methyl orange from complex wastewater. Chin. Chem. Lett. **31**, 539-542. (10.1016/j.cclet.2019.07.037)

[68] Chang L, Ding Y, Li X. 2013 Surface molecular imprinting onto silver microspheres for surface enhanced Raman scattering applications. Biosens. Bioelectron. **50**, 106-110. (10.1016/j.bios.2013.06.002) 23838276

[69] Yang L et al. 2014 Ag–Cu_2_O composite microstructures with tunable Ag contents: synthesis and surface-enhanced (resonance) Raman scattering (SE(R)RS) properties. RSC Adv. **4**, 17 249-17 254. (10.1039/C4RA00675E)

[70] Pham TT, Vu XH, Dien ND, Trang TT, Chi TT, Phuong PH, Nghia NT. 2022 Ag nanoparticles on ZnO nanoplates as a hybrid SERS-active substrate for trace detection of methylene blue. RSC Adv. **12**, 7850-7863. (10.1039/D2RA00620K) 35424719 PMC8982176

[71] Murali DS, Kumar S, Choudhary RJ, Wadikar AD, Jain MK, Subrahmanyam A. 2015 Synthesis of Cu_2_O from CuO thin films: optical and electrical properties. AIP Adv. **5**, 047143. (10.1063/1.4919323)

[72] Wang B, Li R, Zhang Z, Zhang W, Yan X, Wu X, Cheng G, Zheng R. 2017 Novel Au/Cu_2_O multi-shelled porous heterostructures for enhanced efficiency of photoelectrochemical water splitting. J. Mater. Chem. A **5**, 14 415-14 421. (10.1039/C7TA02254A)

[73] Sheng S, Ren Y, Yang S, Wang Q, Sheng P, Zhang X, Liu Y. 2020 Remarkable SERS detection by hybrid Cu_2_O/Ag nanospheres. ACS Omega **5**, 17 703-17 714. (10.1021/acsomega.0c02301) PMC7377325 32715257

[74] Yi Z et al. 2017 Fabrication of well-aligned ZnO@ Ag nanorod arrays with effective charge transfer for surface-enhanced Raman scattering. Surf. Coat. Technol. **324**, 257-263. (10.1016/j.surfcoat.2017.05.084)

